# HGF/MET信号通路在非小细胞肺癌EGFR-TKI耐药性中的研究进展

**DOI:** 10.3779/j.issn.1009-3419.2014.10.08

**Published:** 2014-10-20

**Affiliations:** 233004 蚌埠，蚌埠医学院第一附属医院肿瘤内科 Department of Medical Oncology, the First Affiliated Hospital of Bengbu Medical University, Bengbu 233004, China

**Keywords:** HGF, MET, 肺肿瘤, EGFR酪氨酸激酶抑制剂, 耐药, HGF, MET, Lung neoplasms, EGFR-TKI, Resistance

## Abstract

肺癌是世界上最常见的恶性肿瘤之一，其中非小细胞肺癌约占80%。肝细胞生长因子（hepatocyte growth factor, HGF）/上皮间质转化因子（mesenchymal-epithelial transition factor, MET）信号通路在许多生物学进程中都发挥着多效性影响，然而，在多种类型的肿瘤中都观察到HGF/MET信号通路的异常激活，并且通过生长因子受体和其他致癌性基因受体通路促进细胞增殖和转移。近年来，HGF/MET信号通路的异常激活被认为是对表皮生长因子受体酪氨酸激酶抑制剂（epidermal growth factor receptor tyrosine kinase inhibitor, EGFR-TKI）产生耐药的一个重要原因。本文将重点阐述该通路异常激活与非小细胞肺癌患者EGFR-TKI耐药的联系。

## Abstract (en)

Lung cancer is one of the most common malignant tumors of the world, and non-small cell lung cancer (NSCLC) makes up about 80%. The hepatocyte growth factor (HGF)/mesenchymal-epithelial transition factor (MET) signal pathway has pleiotropic effects on many biological processes. However, aberrant HGF/MET signal pathway activation has been observed in many tumor types, and promotes cellular proliferation and metastasis via growth factor receptors and other oncogenic receptor pathways. In recent years, aberrant HGF/MET signal pathway activation has been considered a key step of resistance to epidermal growth factor receptor tyrosine kinase inhibitor (EGFR-TKI) therapy. This review addresses how aberrant HGF/MET signal pathway activation being associated with drug resistance to EGFR-TKI therapy in NSCLC patients.

表皮生长因子受体酪氨酸激酶抑制剂（epidermal growth factor receptor tyrosine kinase inhibitor, EGFR-TKI）现已成为治疗非小细胞肺癌（non-small cell lung cancer, NSCLC）的常规手段之一，但在临床上这类药物并不适用于所有NSCLC患者，大部分敏感突变的患者在经过大约12个月的初始治疗后都产生了获得性耐药，产生耐药的机制很复杂，其中原发性耐药的机制主要包括EGFR耐药突变、T790突变、*KRAS*突变、*PTEN*缺失和*MET*扩增^[1]^；然而现在研究的更加深入的是获得性耐药，它的机制主要有：*EGFR*的二次突变、下游信号通路的激活、表型转变和*EGFR*突变之外的遗传学改变^[2]^。在众多EGFR-TKI耐药机制中，肝细胞生长因子（hepatocyte growth factor, HGF）/上皮间质转化因子（mesenchymal-epithelial transition factor, MET）信号通路的异常激活在原发性耐药和继发性耐药中都发挥了重要作用，也是近年来研究的热点，研究人员也开发出了许多针对该通路的靶向药物。

## 1 HGF/MET信号通路的成员

### 1.1 HGF

HGF也叫做散射因子，是纤维蛋白溶酶原家族成员，HGF在体内以单链前体被合成，在蛋白水解酶作用下水解成有活性的双链分子，成熟的HGF是由α链和β链经二硫键链接而成的异二聚体，具有活化MET的功能^[3]^，在体内，生理条件下HGF是一种具有多种功能的生物因子，它参与血管生成、组织再生及免疫活性的调节^[4]^。

### 1.2 MET和HGFR

*MET*是一个原癌基因，包含以下结构区：Sema区（Sema domain）、四个IPT区（immunuoglobulin-plexin-transcription domain）、PSI区（Plexins, semaphorins and integrins domain）、JM区（Juxtamembrane domain）、TK区（tyrosine kinase catalytic domain）和TM区（transmembrane domain）。Sema区为配体结合区，JM区有负调节激酶的活性，TK区则包含多个酪氨酸磷酸化位点并具有启动酪氨酸激酶活性的作用^[5]^。

肝细胞生长因子受体（hepatocyte growth factor receptor, HGFR）是MET编码的蛋白质产物^[6]^，是一种跨膜酪氨酸激酶受体，与配体HGF结合后触发受体二聚化，进一步启动下游信号通路^[7]^。HGFR可表达于人体许多器官或者组织的细胞内，包括肝脏、胰脏、前列腺、肾、肌肉和骨髓等^[8]^，正常生理情况下它的活化能激活很多不同的细胞内信号通路，从而影响细胞的增殖、运动、移行、浸润等功能^[9]^。HGFR在多种恶性肿瘤中存在调节异常，在肿瘤细胞的增殖、细胞扩散、血管形成、细胞粘附、细胞侵袭、细胞运动和抗凋亡中发挥着重要作用^[10]^。

## 2 HGF/MET信号通路

HGFR的Sema区与HGF结合后，TK区便发生二聚化和磷酸化，HGFR的羧基末端会募集一些衔接蛋白，如生长因子结合蛋白2（growth factor receptor-bound protein 2, Grb2）、Grb2连接蛋白1（Grb2-associated binding protein, GAB1）和非受体酪氨酸激酶Src，之后Grb2和GAB1可以吸引更多的信号蛋白，包括*Src*基因家族同源区-2（Src homology 2 domain-containing protein, Shc）、PI3K、磷脂酶C（phospholipase C, PLC）、酪氨酸磷酸酶SHP2和信号转导转录激活子3（signal transducer and activator of transcription 3, STAT3），通过这些信号蛋白进一步激活下游几个重要的通路：PI3K-Akt信号通路、Ras-MAPK信号通路和STAT3通路^[11]^。

生理状态下HGF/MET信号通路在胚胎和成人机体内都有表达，在胚胎发育过程中，HGF/MET信号通路在有丝分裂、形态生成等过程中扮演重要角色，在成人体内，该信号途径则在组织损伤后的修复和再生中发挥作用^[12, 13]^。HGF/MET也被认为与血管生成和内皮细胞功能调控有关，在多个模型中，HGF/MET信号激活可以诱导包括血管内皮生成因子（vascular endothelial growth factor, VEGF）在内的血管生成因子，并且与VEGF2协同参与了肿瘤的早期形成^[14, 15]^。HGF/MET信号途径主导的这些功能的一个潜在机制是短暂的上皮间质转化（epithelial-mesenchymal transition, EMT），其特征是上皮分化、细胞扩散和迁移以及细胞外基质降解的缺失，在恶性肿瘤中，对这些进程严格调控能力的丧失导致了侵袭和转移发生^[16, 17]^。

## 3 HGF/MET信号通路异常与NSCLC

HGF/MET信号通路异常激活包括HGF和HGFR过表达、MET扩增和*MET*突变。其中发生率最高的是HGF和HGFR过表达，Tachibana等^[18]^利用FISH和IHC检测了106例肺腺癌患者的HGF和HGFR的表达率分别为17.9%和28.3%，并且在浸润性腺癌中的表达明显强于非浸润性腺癌。最新的研究^[19]^发现在ALK（+）的NSCLC患者中，HGFR的表达水平要明显高于ALK（-）患者。

近年，*MET*基因拷贝数的作用越来越受到人们重视，Park等^[20]^检测了NSCLC患者组织中EGFR和MET的相对拷贝数，发现EGFR和MET在非小细胞肺癌的发生和发展中可能存在相互或者协同作用，并能对非小细胞肺癌患者进行预后评估。此外，有研究者^[21]^分析了213例NSCLC患者的病理切片，发现*MET*基因拷贝数增加是术后NSCLC患者预后不良的独立因素。

MET编码序列点突变的发生率很低，但在许多实体瘤中都有报道，在NSCLC中，*MET*突变可以发生在Sema区、JM区和TK区，Sema区发生的错义突变与MET二聚化有关，且易发生于鳞癌和吸烟患者，JM区的基因突变则与肿瘤发生有关^[22, 23]^。

## 4 HGF/MET信号通路与NSCLC耐药性

### 4.1 原发性耐药

HGF/MET信号通路在NSCLC原发性耐药和继发性耐药中都发挥了重要作用。Yano等^[24]^发现在EGFR-TKI原发性耐药的肺腺癌细胞系中，HGF呈现出了异常的高表达，这提示HGF吉非替尼原发性耐药中扮演着重要角色，HGF表达增加会过度激活MET介导的PI3K-Akt信号通路，降低EGFR-TKI对这种信号级联反应的抑制，与获得性耐药不同，原发性耐药主要是由HGF刺激MET通过GAB1而不是ERBB3的作用激活下游信号通路^[25]^。

Benedettini等^[26]^通过建立的吉非替尼耐药细胞系的MET活化模型，发现如果细胞存在大量MET活化，那么EGFR-TKI对癌细胞的作用将大大受限，即便是敏感突变的细胞也是如此，这也表明MET活化与EGFR-TKI的原发性耐药有着密切关联。最新的一项研究^[27]^发现HGF的存在可导致EGFR酪氨酸激酶的失活，从而引起EGFR-TKI的原发性耐药，同时诱导EGFR与其他的肿瘤相关蛋白相互作用，结论认为应用EGFR-TKI治疗上皮来源肿瘤之前采用HGF/MET抑制剂预处理可防止耐药的发生。

### 4.2 继发性耐药

在亚洲人群中，HGF高表达、T790M突变、*MET*扩增在EGFR-TKI耐药原因中分别占61%、52%和9%，耐药组织中HGF表达水平要明显高于EGFR-TKI敏感组织（*P* < 0.001），一半的耐药肿瘤样本同时存在HGF过表达、T790M突变和*MET*扩增^[28]^。在体外，通过同时抑制HGF高表达、T790M突变和*MET*扩增这三种耐药机制，研究者发现联合EGFR-TKI（WZ4002）和MET-TKI（E7050）能够有效对抗细胞株对厄洛替尼的耐药性^[29]^。在没有*EGFR* T790M和*MET*扩增的配对样本中，治疗后出现耐药组的HGF表达明显高于治疗前的样本（*P*=0.025），这说明HGF是促进药物耐药的一个独立因素，与*MET*原癌基因扩增是相互独立的^[25]^；对耐药患者血液中HGF的检测也得到了一致的结论^[30]^。

最新研究^[31]^表明HGF可诱导敏感肺癌细胞PC-9和H292对吉非替尼耐药，并且HGF刺激MET磷酸化可能是敏感肺癌细胞对吉非替尼耐药的重要机制。研究^[32]^还发现在EGFR-TKI环境下，HGF可以通过促进*MET*扩增增强细胞的耐药性。HGF过表达还与NSCLC患者的不良预后有关，数据显示HGF过表达和低表达患者的5年生存率分别为22.2%和75.0%（*P*=0.259）^[33, 34]^。

在出现EGFR-TKI获得性耐药的NSCLC患者中，*MET*扩增率约为5%-22%^[35]^。Engelman等^[36]^建立了对吉非替尼产生获得性耐药的肺癌细胞株，并对其进行干预，发现*MET*原癌基因的扩增可以通过激活依赖EGFR的ERBB3磷酸化和下游PI3K/AKT通路避开EGFR-TKI的靶点，从而导致NSCLC对EGFR-TKI产生耐药。数据^[37]^显示，在对吉非替尼或厄洛替尼耐药的肺腺癌患者中，有21%的病例出现*MET*扩增，而没有用吉非替尼和厄洛替尼患者的*MET*扩增率只有3%，这提示*MET*扩增与EGFR-TKI耐药有关。虽然*MET*扩增和*EGFR* T790M突变可以同时出现，但T790M和*MET*基因拷贝数呈负相关，表明两者在获得性耐药机制中是互补的关系^[38]^。

## 5 抑制NSCLC中HGF/MET信号通路的研究进展

自从发现HGF/MET信号通路是导致EGFR-TKI耐药的一个重要因素后，人们便不断寻找克服的方法，研发针对HGF/MET信号通路的靶向药物也成为目前靶向治疗研究的一个热点，主要是在以下两个传导水平抑制该通路：第一种是通过抗HGF或者HGFR在配体/受体水平阻断该信号通路，已进入Ⅱ期临床试验的代表药物包括：①抗HGF的单抗：AMG-102（rilotumumab）和AV-299（ficlatuzumab）；②抗HGFR单抗：MetMab（onartuzumab）已经进入了Ⅲ期临床试验，并得到了可喜的结果^[39]^。还有一种通过小分子MET抑制剂可以在酪氨酸激酶区水平抑制该通路。近来，有一大批针对MET酪氨酸激酶区的小分子药物进入临床试验，已经进入临床观察的MET-TKI：Tivantinib（ARQ 197)、Foretinib（XL880）和Cabozantinib（XL184）^[40]^。

虽然针对HGF/MET信号通路的靶向药物层出不穷，但现在学者们普遍认为针对不同耐药机制的联合靶向治疗是克服耐药的最好方法。

## 6 结语

总之，HGF/MET信号通路在人体生理功能中不可或缺，其异常激活在肿瘤发生、发展和靶向治疗耐药方面也发挥着重要作用。随着靶向治疗耐药机制研究的不断深入，很多新的靶向药物已进入临床试验，相信不久的将来，NSCLC患者会有更多更好的靶向治疗选择。
